# Physicochemical quality assessment of four asparaginases

**DOI:** 10.1371/journal.pone.0326106

**Published:** 2025-06-16

**Authors:** Vanessa Radtke, Thorsten König, Christoph Radcke, Ulf Bergmann, Rene Eichler, Katja J. Pohl, Arndt Schnuchel

**Affiliations:** 1 medac Gesellschaft für klinische Spezialpräparate m.b.H., Wedel, Germany; 2 Biosynth GmbH, Berlin, Germany; 3 Proteome Factory AG, Berlin, Germany; 4 Wacker Biotech GmbH, Jena, Germany; University of Nebraska Medical Center, UNITED STATES OF AMERICA

## Abstract

L-Asparaginases (ASNases) are used for the treatment of acute lymphoblastic leukaemia. There are reports of quality problems for some therapeutic asparaginase products, especially those manufactured in middle-income countries. These products may exhibit decreased potency and/or decreased specific activity, or an elevated level of impurities such as host cell proteins. In this study, four different ASNase preparations that were not modified with polyethylene glycol were compared in detail regarding their quality: Spectrila®, Celginase™, Bionase®, and L-Aspase®. Samples were analyzed for protein content, impurities, and enzyme activity. Various chromatographic methods as well as mass spectrometry were used to assess purity and identity. Sample protein content, host cell protein, and enzyme activity showed some results that were out of target range for Celginase™ and Bionase®. These ASNase preparations also showed detectable levels of endotoxins. In gel electrophoresis, additional bands were found for Bionase®. Size exclusion chromatography showed increased high and low molecular weight species for Bionase® and L-Aspase®, and reversed-phase chromatography showed increased hydrophilic and hydrophobic species for Bionase®. In capillary zone electrophoresis, increased retention time for L-Aspase® and increased levels of charge variants for Bionase®, Celginase™, and L-Aspase® were seen. ASNase quality standards are crucial to ensure patient safety and product efficacy, as decreased potency and specific activity may affect efficacy in acute lymphoblastic leukaemia treatment, and increased impurities may affect immunogenicity. Out of four ASNase preparations tested in this study, only Spectrila® did not raise any quality concerns. The other three products exhibited quality problems, rendering them unsuitable according to established quality requirements defined in European and US guidelines for pharmaceutical development of parenteral drug products.

## Introduction

L-Asparaginase (ASNase) is a homo-tetrameric enzyme with a molecular weight of about 140 kDa. ASNases are used for the treatment of acute lymphoblastic leukaemia (ALL) as part of a combination therapy with other antineoplastic reagents, primarily in children. By hydrolyzing extracellular L-asparagine to L-aspartic acid and ammonia, ASNase rapidly and completely depletes plasma L-asparagine. Since asparagine is an essential amino acid for the survival and growth of lymphoblastic cancer cells, its depletion leads to tumor cell apoptosis [1]. In contrast to leukaemic cells, normal cells can synthesize L-asparagine themselves and are therefore much less sensitive to the absence of this amino acid [2]. The bacterial aminohydrolase L-Asparaginase type II from *E. coli* was identified and clinical testing began in 1966 [3]. *E. coli* ASNase in its native form or modified with polyethylene glycol (PEG) is still the most common type of asparaginase used for cancer therapy [4]. While non-recombinant versions of this pharmaceutical product have been marketed since 1978 [3], Spectrila^®^ (medac GmbH) is the first recombinant *E. coli* asparaginase product which has obtained central marketing authorization approval in Europe in 2016. The key improvements of the recombinant L-Asparaginase were a much lower level of aggregates and a very consistent quality level warranted by a state-of-the-art manufacturing process [5].

To decrease immunogenicity and extend plasma half-life and hence lower frequency of application [6], ASNases for human use are often modified with PEG. PEGylated ASNases are the main form of ASNases used in high-income countries. However, low- and middle-income countries rely on more affordable native ASNases.

The number of ALL cases is increasing [7], resulting in a growing global demand for ASNases that has led to shortages [8]. New manufacturers have seen this demand and offer new asparaginase products, thereby creating additional supply and lowering costs. However, there are reports of quality problems for some asparaginase products, especially those manufactured in middle-income countries [4,9]. This may be due to fewer regulatory requirements in the countries where these ASNase preparations are produced. Affected products showed, e.g., decreased potency, specific activity, and/or an elevated level of impurities such as host cell proteins (HCP) [4].

ASNase quality standards are crucial to ensure patient safety and product efficacy. For example, decreased potency and specific activity may affect efficacy in ALL treatment. Impurities, especially HCPs, may increase adverse reactions against the product. Moreover, there have been reports about increased mortality due to the use of low-quality asparaginase products [9,10].

In this study, four different non-PEGylated asparaginase preparations were compared in detail regarding their quality: Spectrila^®^ (medac Gesellschaft für klinische Spezialpräparate m.b.H.), Celginase™ (CELON Laboratories Pvt Ltd.), Bionase^®^ (Zydus Oncosciences, a division of Cadila Healthcare Ltd.), and L-Aspase^®^ (Naprod Life Sciences Pvt Ltd.). Apart from Spectrila^®^, these are products from Indian manufacturers that are marketed mainly in low-income countries.

## Materials and methods

Four asparaginase products from different manufacturers (Table 1) were compared to each other regarding their physicochemical properties by analyzing one lot each side by side. All asparaginase products were available as lyophilizates. Before analysis, the asparaginases were reconstituted in water for injection, which resulted in a final volume of 3.8–4.0 mL depending on the L-asparaginase preparation.

**Table 1:** Overview of tested asparaginases.

Drug Name	Manufacturer	Declared content
Spectrila^®^	medac Gesellschaft für klinische Spezialpräparate m.b.H.	10,000 IU
Celginase™	CELON Laboratories Pvt. Ltd.	10,000 IU
Bionase^®^	Zydus Oncosciences, a division of Cadila Healthcare Ltd.	10,000 IU
L-Aspase^®^	Naprod Life Sciences Pvt. Ltd.	10,000 IU

Asparaginase samples were analyzed for protein content, *E. coli* HCPs, *E. coli* host cell DNA, endotoxins, enzyme activity, isoforms, undesired modifications (i.e., deamidation and oxidation), and protein identity.

### Protein content

Determination of sample protein concentration was performed photometrically by absorption measurement at 278 nm.

### Host cell protein content

*E. coli* HCP content was analyzed using a commercially available enzyme-linked immunosorbent assay (ELISA) kit from Cygnus Technologies (catalogue number F1020). The *E. coli* HCP assay is a two-site immunoenzymetric assay and was performed according to the manufacturer’s instructions.

### Host cell DNA

Residual *E. coli* DNA was determined by using a real-time polymerase chain reaction assay with a commercially available kit from Roche (catalogue number 07 728 735 001). This assay is sensitive and specific for *E. coli* DNA and not subject to detection of human or environmental DNA that might be introduced during sample handling. A commercial kit from Roche was used for DNA extraction (catalogue number 08 146 829 001).

### Endotoxins

Bacterial endotoxins were determined according to Ph. Eur. 2.6.14 (limulus amebocyte lysate test, chromogenic kinetic method, method D).

### Enzyme activity

Activity of asparaginases was determined by Nessler assay [11]. The method is based on the detection of ammonia that is released along with L-aspartic acid from the hydrolysis of L-asparagine by asparaginase.

The asparaginase samples were incubated with L-asparagine in a defined reaction buffer. Subsequently, the samples were incubated with Nessler reagent to form a yellow salt. Absorption was measured at 450 nm.

### SDS-PAGE

Sodium dodecyl sulfate polyacrylamide gel electrophoresis (SDS-PAGE) was performed as reported previously [12]. SDS-PAGE was performed under reducing and non-reducing conditions, by using Invitrogen pre-cast gels. Samples were heated to 70°C for 10 minutes prior to SDS-PAGE. Gels were stained by using the SimplyBlue™ SafeStain kit (Invitrogen) according to the manufacturer’s instructions.

### Size exclusion chromatography

Size exclusion high performance liquid chromatography (SEC) was performed on a Superdex 200 increase column on an Agilent 1260 Infinity II system. A 40 mM potassium phosphate buffer was used as mobile phase (300 mM NaCl, pH 6.5) at a flow rate of 1 mL/min. Protein elution was detected at 220 nm.

### Reversed-phase liquid chromatography

Samples were analyzed by reversed-phase high performance liquid chromatography (RP-HPLC) by using an Asahipak C4P-column on a Waters Alliance e2695 system. Eluent A was HPLC-grade water with 0.1% trifluoroacetic acid (TFA), eluent B was acetonitrile/ water (70/30) with 0.085% TFA. Samples were equilibrated with 10% eluent B, and elution was run from 10% B to 70% B at a flow rate of 0.7 mL/min. Detection of protein elution was performed at 220 nm.

### Capillary zone electrophoresis

Capillary zone electrophoresis (CZE) was performed on a PA 800 plus electrophoresis system (Beckmann Coulter/Sciex) with a tricine-based electrophoresis buffer as reported previously [12].

### Mass spectrometry

Peptide mapping via mass spectrometry was performed as reported previously [12]. The samples were digested with trypsin in solution without reduction and alkylation. The peptides were applied to liquid chromatography-electrospray ionization-tandem mass spectrometry (LC-ESI-MS/MS) analysis with an Agilent 1100 HPLC system coupled with an Orbitrap Velos MS (Thermo Scientific, Bremen, Germany).

Corresponding databases and software were used to determine sequence coverage and identity, namely Mascot MS/MS database [13] and StavroX [14]. The accession number for asparaginase is: Uniprot P00805.

## Results

### Sample protein content, host cell protein, and enzyme activity showed some results that were out of target range

An overview of the sample protein content, contamination with host cell components, endotoxin content, and enzyme activity is shown in Table 2. Protein content was low for Celginase™ and Bionase^®^. Also, both of these ASNases showed elevated levels of HCPs, as detected by ELISA. Enzyme activity was low for Bionase^®^, with only about half of the declared content.

**Table 2:** Overview of test results.

	Spectrila^®^	Celginase™	Bionase^®^	L-Aspase^®^	Target (range)
Protein content	40 mg/vial	36 mg/vial	29 mg/vial	45 mg/vial	40–48 mg/vial
Host cell protein	1.2 ppm	342 ppm	> 1064 ppm	4.9 ppm	≤ 100 ppm
	11.4 ng/mL	3,214 ng/mL	> 8,088 ng/mL	57.8 ng/mL	
Host cell DNA	<LOD	<LOD	<LOD	<LOD	n/a
Endotoxins	< 2.0 IU/vial	2.2 IU/vial	2.8 IU/vial	< 1.9 IU/vial	≤ 10 IU/vial
	< 0.50 IU/mL	0.57 IU/ml	0.73 IU/mL	< 0.50 IU/mL	
Enzyme activity	9,428 IU/vial	8,216 IU/vial	5,062 IU/vial	10,306 IU/vial	80–120% of declared content
	94% of declared content	82% of declared content	51% of declared content	103% of declared content	

LOD, limit of detection [1 pg/mL].

### SDS-PAGE showed additional bands for Bionase^®^

SDS-PAGE was performed under reducing and non-reducing conditions. Due to the conditions during SDS-PAGE it is expected that the homo-tetrameric structure of asparaginase dissociates and only the monomeric subunits can be detected.

**Fig 1:**
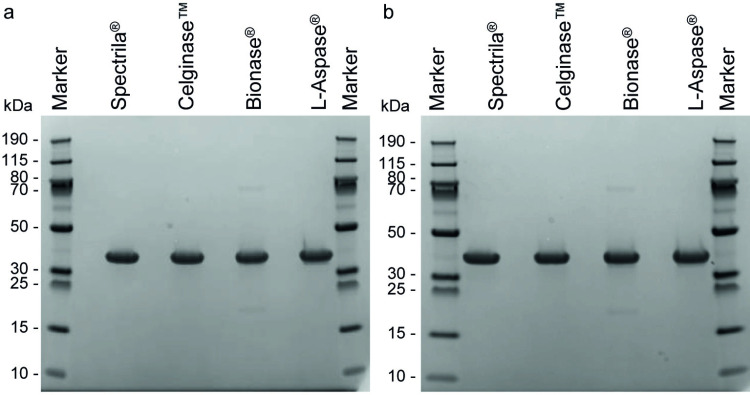
a Non-reducing SDS-PAGE of test samples and **b Reducing SDS-PAGE of test samples.** Molecular weights of the protein markers are indicated in kDa.

In non-reducing SDS-PAGE, the Spectrila^®^, the Celginase™, and the L-Aspase^®^ preparations showed a single prominent band at approx. 37 kDa (Fig 1a) which corresponded well with the molecular weight expected for the asparaginase monomer. In addition to a main band at 37 kDa (approx. 96%), Bionase^®^ showed a high molecular weight (HMW) band at approximately 65 kDa and a low molecular weight (LMW) band at 18 kDa, both with levels of approximately 2% (Fig 1a, Table 3).

**Table 3:** Apparent molecular weights of proteins detected in non-reducing and reducing SDS-PAGE.

Non-reducing SDS-PAGE									
Spectrila^®^		Celginase™		Bionase^®^		L-Aspase^®^		Target	
kDa	%	kDa	%	kDa	%	kDa	%	kDa	%
37	100	37	100	37	96	38	100	Approx. 34–38 (monomer)	≥ 95
				65	2				
				18	2				
**Reducing SDS-PAGE**									
**Spectrila^®^**		**Celginase™**		**Bionase^®^**		**L-Aspase^®^**		**Target**	
**kDa**	**%**	**kDa**	**%**	**kDa**	**%**	**kDa**	**%**	**kDa**	**%**
39	100	39	100	39	96	39	100	Approx. 34–38 (monomer)	≥ 95
				72	2				
				19	2				

The protein bands observed for reducing conditions were very similar to those for non-reducing conditions (Fig 1b, Table 3).

### Size exclusion chromatography showed increased high and low molecular weight species for Bionase^®^ and L-Aspase^®^

**Fig 2:**
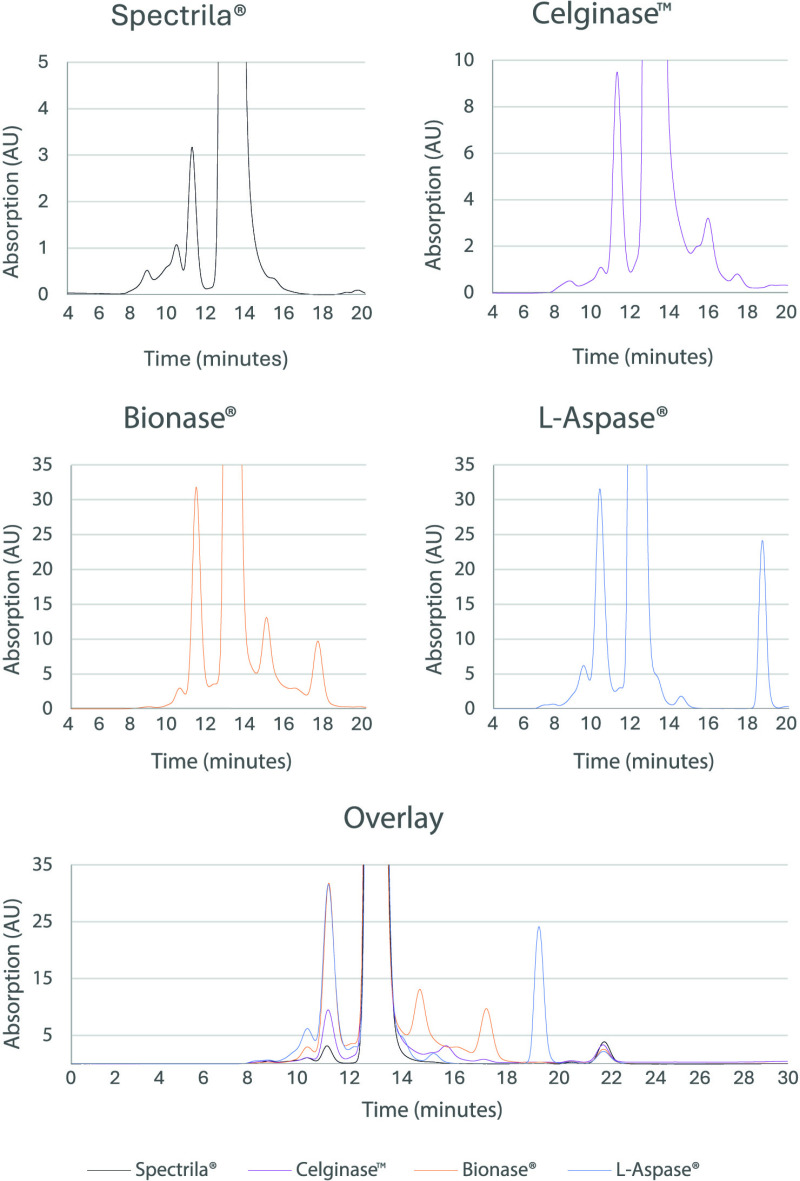
Size exclusion chromatograms of test samples. Zoomed-in view of the peak integration at low signal intensity, and overlay of all four asparaginases. The peaks at 22–23 minutes that were detected for all asparaginases correspond to salt peaks (column dead volume).

HMW and LMW species of asparaginase preparations were analyzed by SEC. The main peak in all test samples eluted with a similar retention at approx. 12.7 minutes, which corresponds to the homo-tetramer (Fig 2). For Spectrila^®^, only minimal levels of HMW species (at lower retention time) and LMW species (at higher retention times) were detected, while the other test items showed elevated HMW and LMW levels. The ratios of the main peak and the sum of LMW or HMW species are summarized in Table 4.

**Table 4:** Relative fraction of asparaginase isoforms as determined by SEC.

	Spectrila^®^	Celginase™	Bionase^®^	L-Aspase^®^	Target
Sum of HMW species [%]	0.7	1.5	5.2	5.8	≤ 3
Main peak [%]	99.3	97.7	91.2	91.1	≥ 95
Sum of LMW species [%]	n.d.	0.8	3.7	3.2	≤ 2

n.d., not detected.

The purities ranged from 91.1% to 99.3% with the highest purities observed for Spectrila^®^ (99.3%) and Celginase™ (97.7%). Markedly lower purities were observed for Bionase^®^ (91.2%) and L-Aspase^®^ (91.1%). In Bionase^®^, the levels of HMW and LMW species were 5.2% and 3.7%, respectively, which is in line with the results obtained for Bionase^®^ by SDS-PAGE. L-Aspase^®^ showed a HMW content of 5.8%, and the levels of LMW species were 3.2%. The LMW signal at 19.6 minutes retention in L-Aspase^®^ differs strikingly in retention time from the main peak and might not be protein-related. In summary, Spectrila^®^ showed the highest purity regarding HMW or LMW variants by SEC.

### Reversed-phase chromatography showed increased hydrophilic and hydrophobic species for Bionase^®^

**Fig 3:**
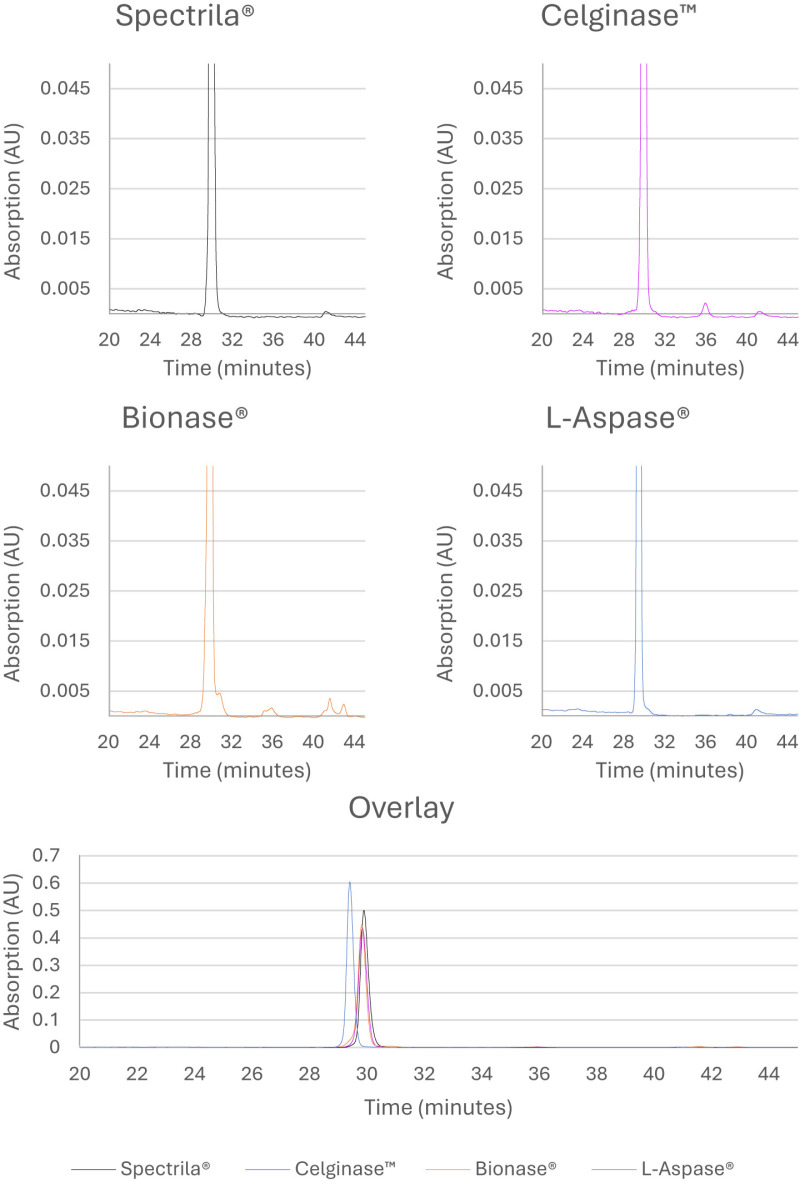
RP-HPLC chromatograms of test samples. Only the part of the chromatograms with the peaks of interest is shown (20–45 minutes). The peaks at 41 minutes that were detected for all asparaginases were also found in blank samples and are not caused by asparaginase samples.

RP-HPLC is particularly suited for the analysis of oxidations and deamidations in proteins as these modifications have an impact on the hydrophobicity of the whole protein. Chromatograms are shown in Fig 3.

Retention of the main species was very similar at approximately 30 minutes for Spectrila^®^, Celginase™, and Bionase^®^. Retention of L-Aspase^®^ was slightly reduced at approximately 29 minutes. The ratios of the main signal, the hydrophobic (later eluting), and the hydrophilic (earlier eluting) species are summarized in Table 5.

**Table 5:** Relative fraction of asparaginase isoforms as determined by RP-HPLC.

	Spectrila^®^	Celginase™	Bionase^®^	L-Aspase^®^	Target
Sum of hydrophilic peaks [%]	n.d.	n.d.	4.1	n.d.	
Main peak [%]	99.4	98.8	92.2	99.2	≥ 98
Sum of hydrophobic peaks [%]	0.6	1.2	3.7	0.8	

n.d., not detected.

No hydrophilic species were detected for Spectrila^®^, Celginase™, and L-Aspase^®^. Bionase^®^ contained a pre-peak shoulder prior to the main peak that was integrated as a hydrophilic peak (4.1%).

The main peaks had ratios of 99.4% for Spectrila^®^, 98.8% for Celginase™, 92.2% for Bionase^®^, and 99.2% for L-Aspase^®^. Hydrophobic species were detected in all preparations at low levels between 0.6% and 1.2%, and at 3.7% for Bionase^®^. In summary, Spectrila^®^ showed the highest purity regarding hydrophobicity-related variants by RP-HPLC.

### Capillary zone electrophoresis showed increased retention time for L-Aspase^®^ and increased levels of charge variants for Bionase^®^, Celginase™, and L-Aspase^®^

**Fig 4:**
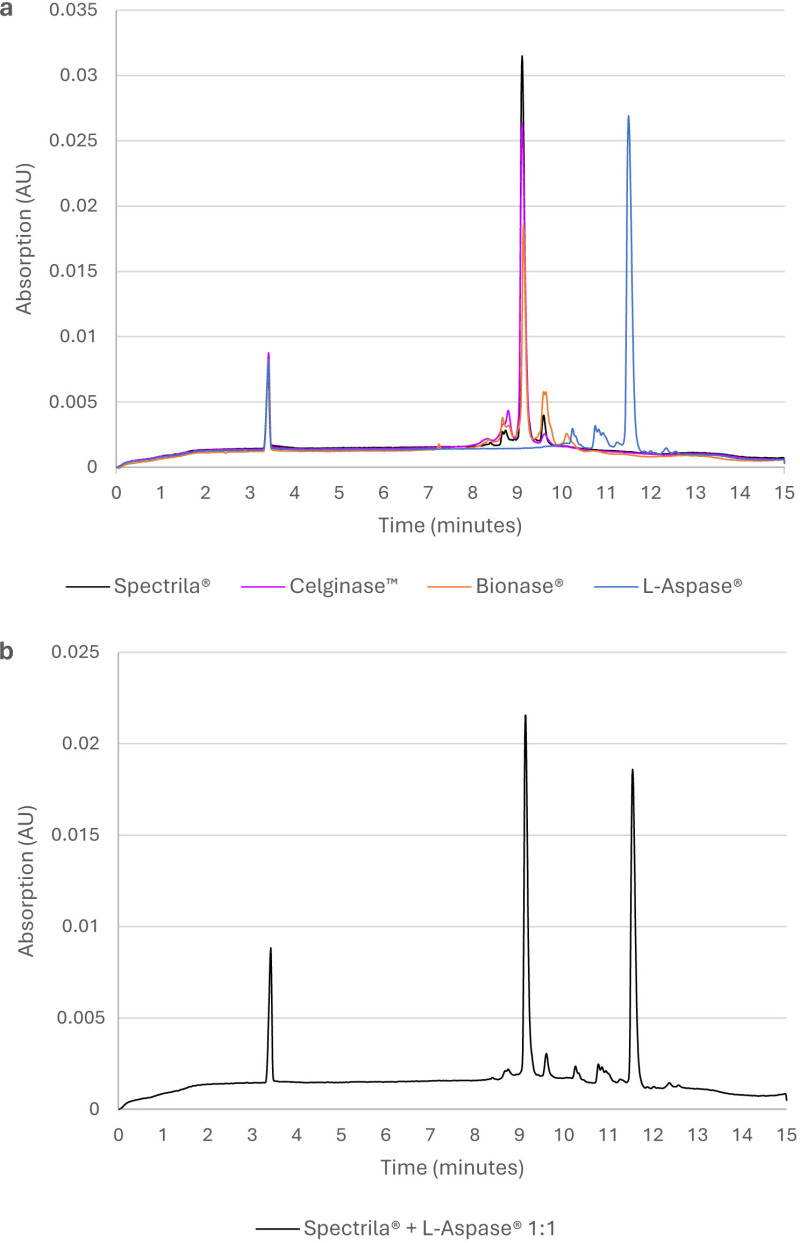
a Overlay of electropherograms and b Electropherogram of 1:1 mixture of L-Aspase^®^ and Spectrila^®^.

Analysis of charge variants by CZE revealed a strikingly increased retention time of the L-Aspase^®^ main peak (11.5 minutes) compared to that of other test items (9.1 minutes each; Fig 4a). To test whether this was due to a matrix effect, we additionally analyzed a 1:1 mixture of the Spectrila® and L-Aspase^®^ sample. CZE of a 1:1 mixture of L-Aspase® and Spectrila^®^ led to the same peak patterns for each species that was observed when L-Aspase^®^ and Spectrila^®^ were analyzed separately (Fig 4b). We found increased levels of charge variants for Bionase^®^, Celginase™, and L-Aspase^®^ (Table 6). For this analysis, we considered the acidic and basic peaks for L-Aspase^®^ in relation to the main L-Aspase^®^ peak (although all peaks appeared shifted compared to the other preparations).

**Table 6:** Relative fraction of asparaginase isoforms as determined by CZE.

	Spectrila^®^	Celginase™	Bionase^®^	L-Aspase^®^	Target
Sum of acidic peaks [%]	7.4	19.7	23.2	22.7	≤ 8
Main peak [%]	85.4	75.7	46.0	75.9	≥ 84
Sum of basic peaks [%]	7.2	4.6	30.9	1.4	≤ 8

### Mass spectrometry yielded high sequence coverage for all tested samples

Tryptic peptides of Spectrila^®^, Celginase™, Bionase^®^, and L-Aspase^®^ were analyzed by HPLC-ESI-MS/MS for identification of peptide sequences. The sequence coverage was 92% for all test samples. Amino acid sequences in disulfide-bridged peptides could not be identified by Mascot MS/MS database search because disulfide linkages are not analyzable by the corresponding software. However, the disulfide-bridged peptides were identified and matched using another software (StavroX) thus increasing the sequence coverage accordingly. Only two very short sequences comprising two or four amino acids, respectively, were not identified. Hence, the overall sequence coverage was 98.2% for Spectrila^®^, Celginase™, and L-Aspase^®^, and 98.8% for Bionase^®^, including the disulfide peptides. Very short peptides can usually not be identified by the corresponding database search or software. Consequently, these were not included in sequence coverage, except for Bionase^®^, for which two more amino acids were identified via a peptide with missed tryptic cleavages that added to the sequence coverage (hence the higher coverage for Bionase^®^).

## Discussion

ASNase products, which are used for ALL treatment, may be subject to differences in quality parameters such as potency or purity. These quality parameters are important, since ASNase integrity and purity may affect its efficacy and safety in ALL treatment. For example, HCPs in ASNase preparations can lead to increased immunogenicity [15,16], potentially leading to adverse events and treatment discontinuation.

Here, we compared the quality of four ASNase preparations containing non-PEGylated ASNase, which is primarily used in middle- and low-income countries. We detected several quality problems. There was low protein content, HCP contamination, and detectable levels of endotoxin in Bionase^®^ and Celginase™, low enzyme activity of Bionase^®^, additional protein bands in SDS-PAGE of Bionase^®^, HMW and LMW species for Bionase^®^ and L-Aspase^®^. Moreover, we detected increased hydrophilic and hydrophobic content in Bionase^®^, and increased retention time in CZE for L-Aspase^®^. CZE also revealed increased acidic content in Celginase™, Bionase^®^, and L-Aspase^®^, as well as increased basic content in Bionase^®^.

L-Aspase^®^ showed marked differences in retention time by CZE (indicating charge variants) and by RP-HPLC (indicating hydrophobicity-related variants). It is possible that mutations or side chain modifications are present that result in shifts of isoelectric point and hydrophobicity. Alternatively, additional amino acids might be present – even multiple additional amino acids. However, *N*-terminal sequencing did not reveal any mutations or additional amino acids (data not shown). Furthermore, no mutations, side chain modifications or additional amino acids were detected by mass spectrometry. However, they may have been present in positions not covered by peptide mapping. It is unlikely that a matrix effect is responsible for the observed shifts, as the CZE of a 1:1 mixture of L-Aspase^®^ and Spectrila^®^ led to the same peak pattern for each asparaginase that was observed when L-Aspase^®^ and Spectrila^®^ were analyzed separately. Furthermore, an isoelectric focusing/charge variant analysis with and without prior dialysis did not show any differences (data not shown). Also, the presence of a non-covalent binder cannot be excluded. However, the hypothetical binder would not dissociate upon dialysis and would not affect asparaginase activity. Consequently, the presence of covalent modifications seems more likely to us.

Taken together, while Spectrila^®^ met all target ranges in the tests conducted during this study, all three biogeneric products were outside the range of at least one test, revealing quality problems like impurities. This raises concerns about possible impacts on the effectiveness and safety of these products.

ASNase products manufactured in low- and middle-income countries have previously been reported to show quality problems. For example, in a review by Qin et al., the authors summarized results of tests which assessed ASNases for potency, specific activity, purity and HCP content. Out of 18 ASNase products, only 8 were within the specified potency range, 5/15 showed sufficient specific activity, 7/17 were acceptably pure, and only 3/16 showed acceptable HCP levels [4]. This highlights the need for thorough testing of ASNase preparations. The authors concluded that ASNases manufactured in high-income countries were of overall better quality than those from middle-income countries [4], which is in line with our results.

ASNase quality may affect its in vivo activity in patients and patient outcomes [9]. In Brazil, country-wide ASNase supply was changed from Aginasa^®^ to Leuginase^®^ in 2017 [17]. Michalowsky and colleagues found that after the change, product-associated 3-year overall survival dropped from 91.8% to 83.8% (p = 0.003) and event-free survival dropped from 84.8% to 76.1% (p = 0.008) [10]. Tests showed that Leuginase^®^ exhibited reduced bioavailability in mice, and it was found to be contaminated with substantial amounts of HCP [18]. This case, along with the fact that ASNase formulations of varying quality are still widely in use, has raised concerns and has led to a call for regulatory institutions to guarantee the application of strict quality standards [19].

## Conclusions

ASNase quality monitoring and stringent regulations for their application in ALL treatment worldwide are important, since the use of low-quality ASNases may affect patient outcomes. Out of four ASNases tested in this study, only Spectrila^®^ did not raise any quality concerns. The other three products tested exhibited one or more quality defects rendering them unsuitable according to established and required quality standards defined in European and US guidelines for pharmaceutical development of parenteral drug products.

While two products failed to meet several quality criteria, one product showed a strikingly different charge profile in several assays. However, within the scope of this study it was not possible to identify the root cause of this untypical behavior. The impact on efficacy and safety cannot be assessed.

In conclusion, this investigation showed that among the four ASNase products tested side-by-side only Spectrila^®^ exhibited no quality defects and thus represents a safe and efficacious pharmaceutical product for treatment of ALL. Based on these results, it becomes apparent that ALL patients in low- and middle-income countries are at risk of receiving low-quality and even worse ineffective treatment from medicines marketed in these countries. Rigorous quality monitoring of the available medicines in these markets is proposed to be implemented to warrant adequate and effective treatment of ALL patients.

## Supporting information

S1 Fig Raw Data SDS-PAGE. (ZIP)

S2 Fig Raw Data SEC. (ZIP)

S3 Fig Raw Data RP-HPLC. (ZIP)

S4 Fig Raw Data CZE. (ZIP)
