# Supplementary figures and images for: Physicochemical quality assessment of four asparaginases
Source: PLoS One. 2025 Jun 16;20(6):e0326106. doi: 10.1371/journal.pone.0326106 (PMC12169553; doi:10.1371/journal.pone.0326106)

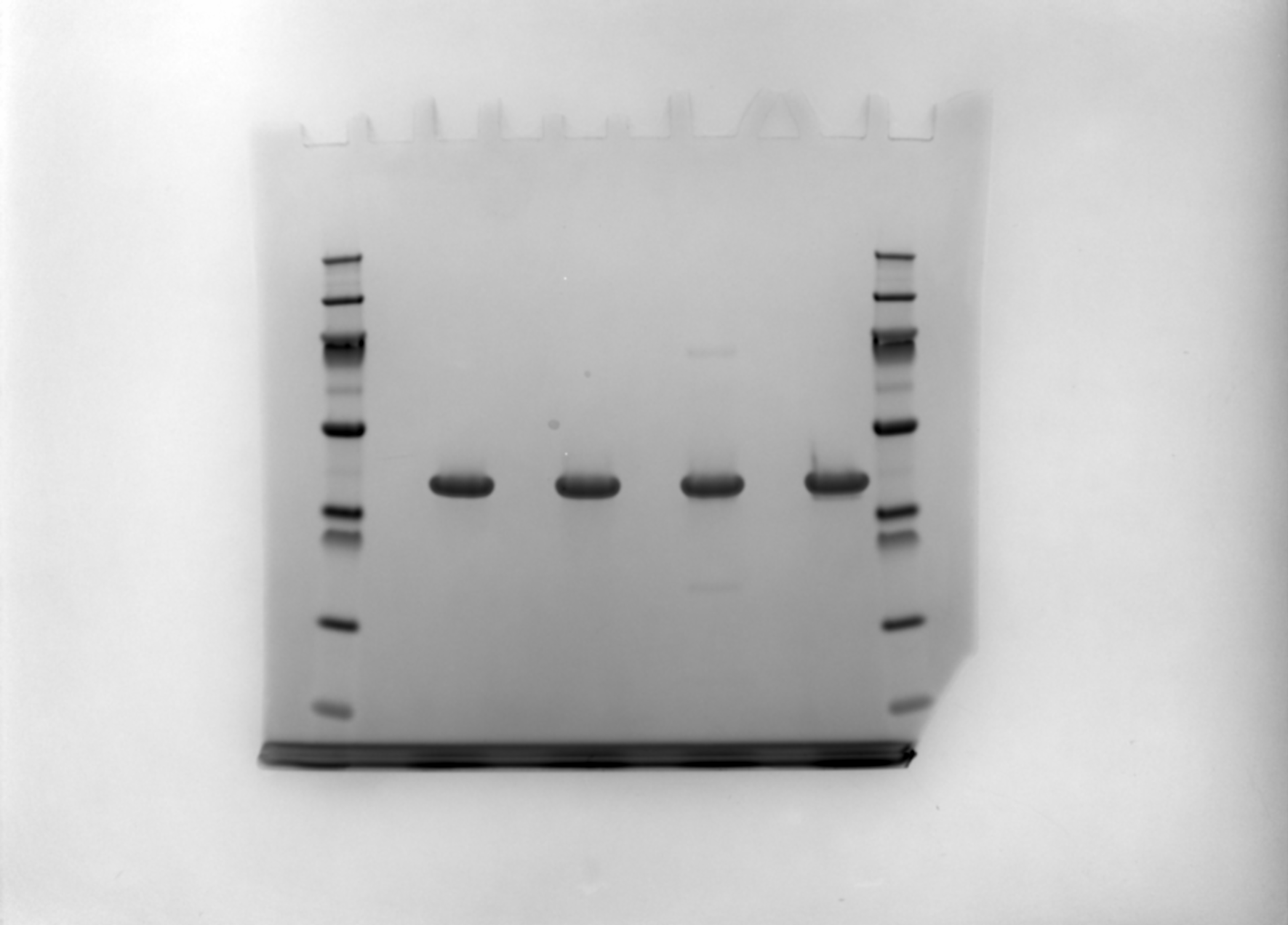

Supplement: S1 Fig — (ZIP) [file pone.0326106.s001.zip › S1 Fig1 Raw Data SDS-PAGE non-reducing.tif]

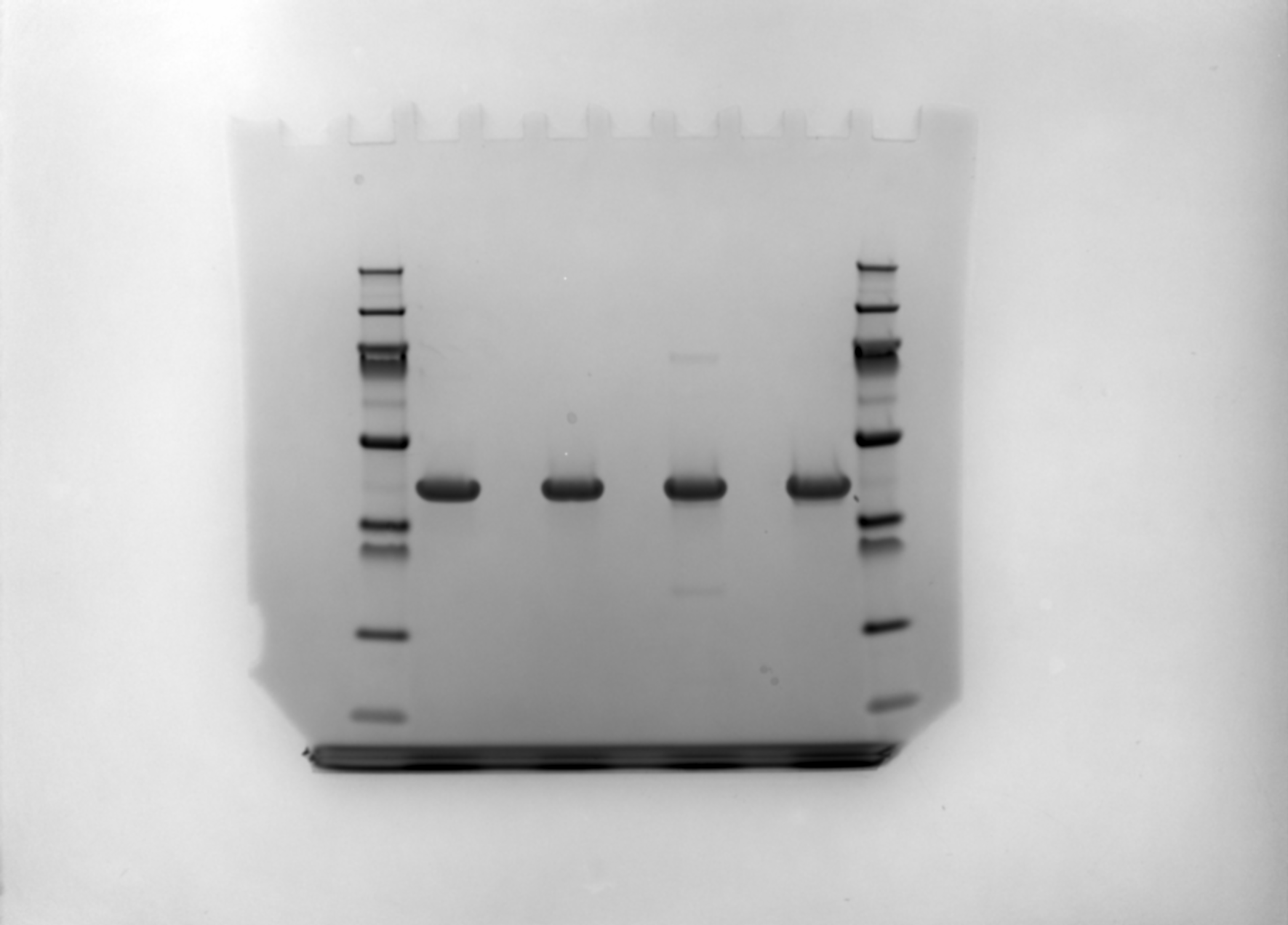

Supplement: S1 Fig — (ZIP) [file pone.0326106.s001.zip › S1 Fig1 Raw Data SDS-PAGE reducing.tif]
